# H-TEX-mediated signaling between hepatocellular carcinoma cells and macrophages and exosome-targeted therapy for hepatocellular carcinoma

**DOI:** 10.3389/fimmu.2022.997726

**Published:** 2022-10-13

**Authors:** Sihang Yu, Lei Zhou, Jiaying Fu, Long Xu, Buhan Liu, Yuanxin Zhao, Jian Wang, Xiaoyu Yan, Jing Su

**Affiliations:** ^1^ Department of Pathophysiology, College of Basic Medical Sciences, Jilin University, Changchun, China; ^2^ Department of Pathology, Affiliated Hospital to Changchun University of Chinese Medicine, Changchun, Jilin, China

**Keywords:** exosomes, hepatocellular carcinoma, liver cancer, hypoxia, TAM, macrophage, therapy, drug resistance

## Abstract

There is increasing evidence for the key role of the immune microenvironment in the occurrence and development of hepatocellular carcinoma. As an important component of the immune microenvironment, the polarization state and function of macrophages determine the maintenance of the immunosuppressive tumor microenvironment. Hepatocellular carcinoma tumor-derived exosomes, as information carriers, regulate the physiological state of cells in the microenvironment and control cancer progression. In this review, we focus on the role of the exosome content in disease outcomes at different stages in the progression of hepatitis B virus/hepatitis C virus-induced hepatocellular carcinoma. We also explore the mechanism by which macrophages contribute to the formation of hepatocellular carcinoma and summarize the regulation of macrophage functions by the heterogeneity of exosome loading in liver cancer. Finally, with the rise of exosome modification in immunotherapy research on hepatocellular carcinoma, we summarize the application prospects of exosome-based targeted drug delivery.

## Introduction

Liver cancer has a 5-year relative survival rate of only 20% (1). Liver cancer caused by chronic infection with hepatitis B virus (HBV) and hepatitis C virus (HCV) accounts for approximately 50%–80% of cases (2). Other risk factors include aflatoxin exposure, tobacco and alcohol use, non-alcoholic fatty liver disease, obesity, and diabetes. The distribution of these risk factors varies according to the population, time, and region (1, 3). Therefore, as liver cancer is a chronic inflammation-related cancer, it is crucial to study the role of exosomes during diseaseprogression and in the immune microenvironment.

Macrophages are abundant in the liver and are essential cells in the tumor microenvironment (TME) in liver cancer. During the initial stages of liver cancer at the time of niche formation, hepatic macrophages display an inflammatory phenotype, namely, the M1 type; these cells damage neighboring cells by continuous secretion of reactive oxygen species. In solid tumors that successfully escape immune surveillance, macrophages disproportionately differentiate into the M2 phenotype with anti-inflammatory activity, that is, tumor-associated macrophages (TAMs), which have proangiogenic, matrix remodeling, distal metastasis, and immunosuppressive effects (4). The recruitment of hepatic macrophages in human liver cancer is correlated with disease progression and a poor prognosis (5). Hepatoma cells also play crucial regulatory roles in macrophage proliferation and differentiation during tumor progression (6).

Hypoxia has become one of the most intensively studied features of the TME (7). In this context, in addition to directly secreting cytokines (8), exosome-mediated communication between tumor cells and the stroma is considered an important step in remodeling the TME (9). Multiple studies have demonstrated the adaptive tuning of extracellular vesicle (EV) secretion and contents of liver cancer cells during progression, providing a basis for subsequent remodeling of the surrounding niche (10, 11) and, specifically, for altering TAMs.

In this review, we summarize the roles of exosomes and macrophages during the progression of viral hepatitis to liver cancer, including but not limited to the effects of exosomal contents on macrophages and exosome-based therapeutic prospects for liver cancer.

## Involvement of exosomes in hepatocarcinogenesis

Analyses of liver cancer progression associated with chronic inflammation are still needed. Hepatocyte exosomes function as messengers in the formation and evolution of the liver cancer niche (12). Exosomes are released into the intercellular space or enter the hepatic microvasculature to participate in intercellular signal communication or material transport, thereby regulating the pathophysiological state of the liver (13).

In the physiological state, liver parenchymal cells, which make up approximately 80% of the liver volume, secrete exosomes loaded with neutral ceramidase and sphingosine kinase 2 (SK2), which are recognized by recipient hepatocytes and upregulate sphingosine-1-phosphate (S1P) production by target cells, thereby promoting hepatocyte repair and regeneration (14).

**Figure 1:**
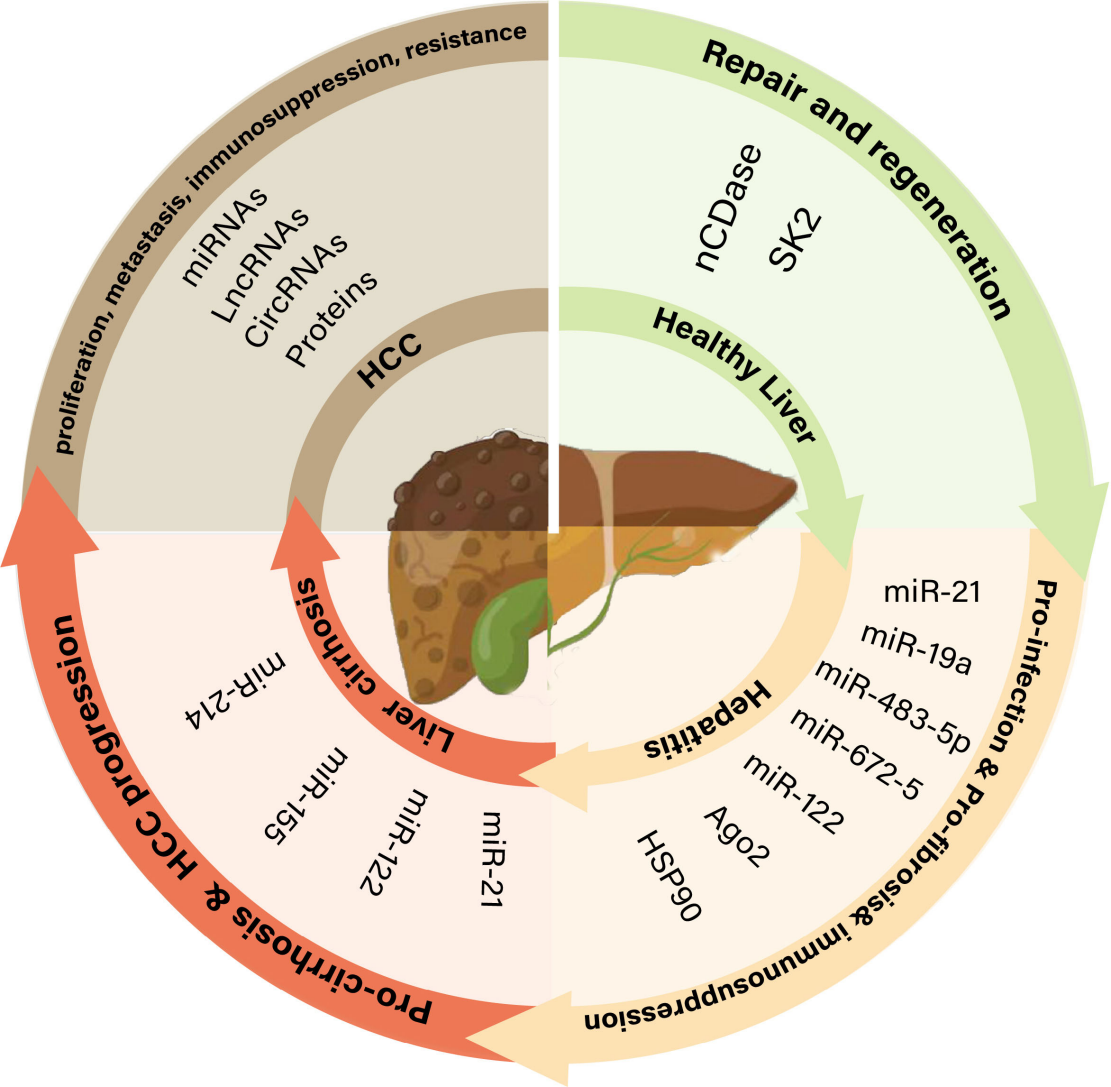
The role of exosomes in different pathophysiological states of the liver. nCDase, neutral ceramidase; SK2, sphingosine kinase 2; Ago2, argonaute-2; Hsp90, heat shock protein 90; lncRNAs, long non-coding RNAs; miRNAs, MicroRNAs; circRNAs, Circular RNAs.

In some pathological conditions, liver parenchymal cells, hepatic stellate cells (HSCs), and Kupffer cells (KCs) are the main donor and recipient cells of exosomes and are associated with hepatitis, cirrhosis, and liver cancer (15). The hepatocyte exosomal cargo plays diverse roles in the microenvironment during liver cancer progression (**Figure 1**).

### Viral Hepatitis

Hepatitis caused by HBV/HCV infection is one of the main causes of hepatocellular carcinoma (HCC) (2). Exosomes secreted from hepatocytes infected with HCV carry virus-derived Ago2, heat shock protein 90 (Hsp90), and miR-122, which mediate the stable transmission of HCV in the liver (16–18). Exosome-mediated viral transport helps the virus evade immune system surveillance. MicroRNAs (miRNAs) released from virus-infected hepatocytes inhibit natural killer (NK) cell proliferation and survival and facilitate the evasion of host innate immunity (19). Exosomes containing HCV RNA reduce Toll-like receptor 3 (TLR3) activation and interfere with antiviral interferon-stimulated gene activation (20). T-cell immunoglobulin and mucin domain-containing molecule 3 (TIM-3)/galectin 9 (Gal-9) in exosomes increase HCV-infected hepatocytes, affect monocyte differentiation, and inhibit the immune response (21).

### Cirrhosis

During liver cirrhosis, HSCs along with other cells of the liver parenchyma (liver sinusoidal endothelial cells and KCs) play important roles in the development and progression of liver fibrosis (22). Exosomes released by injured hepatocytes are internalized by stellate cells, leading to phenotypic switching of quiescent stellate cells. HSC activation is a major driver of the initiation, progression, and resolution of liver fibrosis (23).

Exosomes released from injured hepatic stellate structures contain abundant fibrotic components that promote fibrosis *via* multiple pathways, such as by stimulating fibroblasts and myofibroblasts to produce collagen from the bone marrow and portal fibrocytes. Connective tissue growth factor (CTGF), a multifunctional heparin-binding glycoprotein, contributes to the promotion of multiple fibrotic processes (24). CTGF, which is widely expressed in activated HSC-derived exosomes, regulates the activation and migration of HSCs and immune responses, whereas exosomes produced by quiescent HSCs are enriched in miR-214 and twist, attenuating the profibrotic function of activated HSCs (25, 26). Exosomes derived from liver sinusoidal endothelial cells regulate the migratory capacity of HSCs *via* adhesion.

### Hepatocellular Carcinoma

HCC is a common malignancy with poor overall survival. The main risk factors for HCC include viral hepatitis, excessive alcohol consumption, and smoking. However, the pathogenesis of HCC is complicated and difficult to determine. Extensive evidence suggests that exosomes derived from cells carry tumor-specific markers, which mediate intercellular communication between cancer cell populations and promote the migration and invasion of recipient cells (27). For non-immune cells, HCC exosomes regulate tumor niche formation by promoting tumor-associated fibroblast transformation and angiogenesis by altering the endothelial vascular phenotype (28, 29). Liver cancer exosomes mainly mediate tumor cell immune escape by inhibiting their maintenance and proliferation, promoting phenotypic transformation, and blocking functional activation (30).

These effects promoting HCC progression depend on proteins and non-coding RNAs (ncRNAs) in exosomes. They are transferred by exosomes and participate in the communication between HCC cells and targeted cells in the TME, thereby affecting tumor angiogenesis, metastasis, and drug and radiotherapy resistance. Therefore, we summarized the current research status of proteins and ncRNAs in HCC exosomes to further emphasize the potential value of these abnormally expressed exosome molecules in HCC as biomarkers for the diagnosis, prognosis, and treatment of HCC (**Table 1**).

**Table 1:** Effects of exosome contents on hepatocellular carcinoma.

Contents	Mechanism	Function	References
**Proteins**			
LOXL4	Activation of FAK/SRC pathway alters cell matrix adhesion and migration ability	Promotes migration and angiogenesis	(31)
GOLM1	Activated glycogen synthase kinase-3 β / MMPs (GSK-3 β/MMPs) of the recipient cells signaling axis	Accelerates cell proliferation and migration	(32)
S100A4	Activation of OPN transcription by STAT3 phosphorylation	Promotes tumor metastasis	(33)
HMGB1	Activation of the TLR-MAPK pathway	Promotes TIM-1(+) B-cell proliferation and inhibits CD8(+) T-cell activity	(34)
SMAD3	Enhanced TGF-β-Smad3-ROS signaling	Promotes proliferation and adhesion	(35)
ENO1	Upregulation of integrin αs6β4 expression	Activates the FAK/Src-p38MAPK pathway to promote the growth and metastasis of HCC cells	(36)
CLEC3B	Promotes the phosphorylation of AMPK, thereby decreasing the expression of VEGF	Attenuates migration and invasion of recipient cells and relieves angiogenesis	(37)
CHI3L1	Activation of MAPK and Akt signaling pathways	Promotes tumor metastasis	(38)
EIF3C	Activation of S100A11 expression	Promotes angiogenesis and tumor development	(39)
**miRNAs**			
miR150	Promotes vascular endothelial growth factor (VEGF) secretion in TAMs	Promotes tumorigenesis	(40)
miR-23a-3p	Upregulation of PD-L1 expression in macrophages *via* STAT3 signaling pathway	Attenuates the anti-HCC immune response	(41)
miR-32-5p	Inhibits PTEN and activates the PI3K/Akt pathway	Induction of multidrug resistance by angiogenesis and EMT	(42)
miR-1247-3p	Downregulation of B4GALT3 and activation β 1-integrin/NF-κB axis	Promotes tumor status, EMT, chemoresistance, tumorigenicity, and metastasis	(43)
miR-638	By downregulating the expression of VE-cadherin and ZO-1 in endothelial cells	Promotes vascular permeability	(44)
miR-27a-3p	By regulating thioredoxin-interacting protein (TXNIP)	Promotes the stemness of liver cancer	(45)
miR-125b	Disrupted TGF-β1-induced epithelial–mesenchymal transition and TGF-β1/SMAD signaling pathway	Antimetastatic effect	(46)
miR-15a-5p	Inhibits PD1 expression in CD8+ T cells	Inhibits the development of HCC	(47)
miR-210	Entry into endothelial cells inhibits SMAD4 and STAT6	Promotes tumor angiogenesis	(48)
miR-93	Inhibits CDKN1A, TP53INP1, and TIMP2	Promotes proliferation and invasion	(49)
miR-374a-5p	Possibly by regulating GADD45A	Promotes proliferation, migration, and invasion of HCC cells	(50)
miR-92a-3p	By inhibiting PTEN and activating the Akt/Snail signaling pathway	Promotes EMT	(51)
miR-320a	Inhibit PBX3/ERK1/2/CDK2 axis	Inhibits proliferation and metastatic ability	(52)
miR-21	Inhibit PTEN, upregulate PDK1/AKT pathway	Transforms normal hematopoietic stem cells into cancer-associated fibroblasts	(53)
miR-451a	Targeting LPIN1 regulates tumor cell apoptosis and angiogenesis	Inhibits hepatocellular tumorigenesis	(54)
**lncRNAs**			
TUC339	May be involved in cytokine receptor signaling pathway and CXCR chemokine receptor-binding pathway	Promotes macrophage polarization to M2 (IL-4) phenotype	(55)
lncRNA H19	By upregulating the miR-520a-3p/LIMK1 axis	Promotes the proliferation, migration, and invasion of HCC cells after propofol treatment and inhibits the apoptosis of HCC cells	(56)
SENP3-EIF4A1	Regulation of ZFP36 expression by competitive binding to miR-9-5p	Able to inhibit tumor growth *in vivo*	(57)
FAL1	Upregulation of ZEB1 and AFP by inhibiting miR-1236	Promotes proliferation and migration	(58)
ASMTL-AS1	by activating the YAP signaling pathway	Accelerates tumor progression	(59)
**circRNAs**			
circ-DB	Enhances the expression of USP7 and Cyclin A2 by inhibiting the expression of miR-34a	Promotes tumor growth, inhibits DNA damage	(60)
circ-PTGR1	Activation of MET by interaction with miR-449a	Promotes migration, invasion, and metastasis	(61)
circ-UHRF1	Suppresses NK cell function by degrading miR-449c-5p and upregulating TIM-3 expression	Promotes immunosuppression	(62)
circ-0051443	By upregulating BAK1	Promotes apoptosis and inhibits cell cycle	(63)
circ-CMTM3	Promotes angiogenesis by regulating the miR-3619-5p/SOX9 axis	Promotes HCC tumorigenesis	(64)
circ-TMEM45A	By upregulating the miR-665/IGF2 axis	Promotes the progression of HCC	(65)

LOXL4, Lysyl Oxidase Like 4; GOLM1, Golgi membrane protein 1; A4S100A4, S100 Calcium Binding Protein; OPN, Osteopontin; HMGB1, High mobility group box 1; TLR, toll-liked receptor; MAPK, mitogen-activated protein kinase; Smad3, mothers against decapentaplegic family member3; TGF-β, Transforming growth factor beta ; ENO1, Enolase 1 ; FAK, focal adhesion kinase ; CLEC3B, C-Type Lectin Domain Family 3 Member B ; AMPK, AMP-activated protein kinase ; VEGF, Vascular endothelial growth factor; CHI3L1, Chitinase-3-like protein 1; EIF3C, Eukaryotic Translation Initiation Factor 3 Subunit C; S100A11, S100 Calcium Binding Protein A11; PD-L1, Programmed death-ligand 1; PTEN, Phosphatase and tensin homolog ; B4GALT3, Beta-1,4-Galactosyltransferase 3; ZO-1, zonula occluden-1; Smad4, mothers against decapentaplegic family member4; STAT6, Signal transducer and activator of transcription 6; CDKN1A, Cyclin Dependent Kinase Inhibitor 1A ; TIMP2, Tissue inhibitor of metalloproteinases 2; EMT, Epithelial–mesenchymal transition; GADD45A, Growth Arrest and DNA Damage Inducible Alpha; PBX3, pre-leukemia transcription factor 3; ERK, extracellular signal‑regulated protein kinase ; CDK2, Cyclin Dependent Kinase 2; PDK1, Pyruvate Dehydrogenase Kinase 1; LPIN1, phosphatidic acid phosphohydrolase1; CXCR, C-X-C Motif Chemokine Receptor; LIMK1, LIM Domain Kinase 1; ZFP36, zinc finger protein 36 homolog ; ZEB1, Zinc Finger E-Box Binding Homeobox 1; AFP, Alpha-Fetoprotein ; YAP, Yes-associated protein ; USP7, Ubiquitin Specific Peptidase 7; MET, mesenchymal-epithelial transition; BAK1, BCL2 Antagonist/Killer 1; SOX9, SRY-Box Transcription Factor 9; IGF2, Insulin Like Growth Factor 2.

## Hypoxia promotes the production and release of exosomes

HCC is a hypermetabolic tumor of the digestive system. Based on the high rate of cell proliferation, the altered blood supply system participates in the exchange of substances within the tumor (66). Therefore, hypoxic signals contribute to liver cancer formation, proliferation, and metastasis (67). In addition to the adaptive changes in cellular components within the TME in response to hypoxia, hepatoma cells transmit post-hypoxic regulatory signals to other cells by secreting EVs (68). Cancer cells with different phenotypes communicate *via* exosomes to complete the phenotypic transformation and promote the progression of liver cancer (69). For example, exosomes from highly metastatic MHCC97H cells can communicate with less metastatic HCC cells, increasing their migration, chemotaxis, and invasion (70). Similarly, the EVs of cisplatin-resistant non-small-cell lung cancer cell lines secreted pyruvate kinase M2 (PKM2) under hypoxic conditions. The phagocytosis of these EVs by cisplatin-sensitive non-small-cell lung cancer cell lines induced decreased sensitivity to cisplatin (71).

The adaptive response of tumor cells to hypoxia is mostly regulated by hypoxia-inducible factor 1 (HIF1). HIF1α/2α is also highly expressed in liver cancer (72). Under normoxia, the two proline residues of the HIF-1/2α subunit are hydroxylated by prolyl hydroxylase domain (PHD) enzymes, promoting binding to von Hippel–Lindau (VHL), which mediates the degradation of the hydroxylated HIF-1/2α subunit *via* the ubiquitin-proteasome pathway. However, under hypoxic conditions, the generation and release of EVs are regulated by HIF. During EV biogenesis, RAS superfamily proteins (RABs) are involved in the formation and fusion of membrane buds, and HIF can directly affect the RAS. That is, under hypoxic conditions, HIF is activated to promote the transcription of RABs and finally promote the generation and secretion of exosomes (73, 74). HIF can promote the expression and activation of a series of cell surface receptors, such as epidermal growth factor receptor, glucose transporter receptor, and transferrin receptor, and promote cell internalization and endocytosis (75).

The mechanism by which EV contents (nucleic acids, proteins, etc.) are specifically sorted under hypoxia remains unclear. This process is related to endosomal sorting complex required for transport (ESCRT) complexes and ceramides and may be related to posttranslational processes (76). This modified protein complex is closely related to ubiquitin-like 3 (UBL3)/membrane-anchored Ub-fold protein (MUB). In models of lung injury, proteins and peptides in vesicles were more ubiquitinated under hypoxia (77). This indicates that ubiquitination regulates the loading process of exosome contents under hypoxia.

In addition, HIF1-independent regulation of adaptive responses to hypoxia has been reported, such as phosphoinositide 3-kinase (PI3K), serine-threonine kinase (AKT), mammalian target of rapamycin (mTOR), Nuclear factor kappa-light-chain-enhancer of activated B cells (NF-κB) Rab-GTPase, Wnt/β-catenin, mitogen-activated protein kinases, and oxidative stress (78).

## Hepatocellular Carcinoma (HCC)-associated macrophages

### Origin and function of macrophages

Under physiological conditions, the liver has a rich blood supply and abundant innate immune cells (such as KCs, NK cells, and T cells). Resident macrophages in the liver are mainly composed of KCs and monocyte-derived macrophages (79). In healthy liver, KCs are the main resident hepatic macrophages. KCs are generally believed to have originated from yolk sac-derived colony-stimulating factor 1 receptor (CSF1R) + erythroid/myeloid progenitors (EMPs), which are present in the fetal liver during embryogenesis. They can maintain liver homeostasis by removing metabolic waste and cell debris, regulating cholesterol homeostasis, maintaining iron homeostasis and iron cycling, mediating immune responses, and promoting immune tolerance (80). Some circulation-derived monocyte-macrophage populations recognize liver-invading bacteria and recruit neutrophils. In the human liver, hepatic macrophages consist of CD68+macrophage receptor with collagenous structure (MARCO)+KCs, CD68+MARCO-macrophages, and CD14+monocytes. CD68+MARCO+-KCs usually overexpress immune tolerance-related genes and have anti-inflammatory effects, and CD68+MARCO-macrophages and CD14+monocytes have pro-inflammatory effects (5).

From the progression of chronic hepatitis to fully developed tumors, there is a high degree of heterogeneity in the intratumoral microenvironment, with a highly invasive anterior and middle hypoxic and necrotic areas and tumor cells with high and low proliferation (81). There are also different TAM phenotypes in liver cancer, and research on the classification of TAMs and their heterogeneity is still in its infancy (82). In short, TAMs are collections of macrophages, including infiltrating and resident macrophages, originating from various cellular sources. TAM polarization shows plasticity, and cells can exhibit either phenotype. Several studies have provided evidence that the acquisition of an M2-like polarized macrophage phenotype by TAMs promotes tumor progression by promoting angiogenesis, immunosuppression, and growth factor secretion, ultimately leading to metastasis (83).

TAMs secrete excessive proangiogenic factors [e.g., vascular endothelial growth factor (VEGF), platelet-derived growth factor, and transforming growth factor beta (TGFB)] and cell proliferation-stimulating factors (e.g., Interleukin(IL)-1β, IL-6, chemokine (C-C motif) ligand 2 (CCL2), tumor necrosis factor, and VEGF), which strongly promote tumor growth and development (84, 85).

### Mechanisms underlying macrophage uptake of exosomes

When exosomal vesicles come in contact with the surface of macrophages, they trigger a functional response (e.g., proliferation and differentiation) *via* membrane surface ligand–receptor recognition signals and/or transport of their contents into the cell, antigen presentation, etc. (86).

Macrophages are initially recognized by protein receptors and adhesion molecules (e.g., tetraspanins, integrins, proteoglycans, and lectins) on the exosome surface. Exosomes are then taken up by activating cell membrane-expressed receptors, fusion with the macrophage plasma membrane, or endocytosis (87). The final contents are delivered to macrophages to exert biological functions. An increasing number of studies have evaluated the mechanism underlying the uptake. For example, exosomes derived from pancreatic cancer preferentially bind to F4/80+ and CD11b+ KCs in the liver, which is promoted by intercellular adhesion molecules and CD11b ligands (88). A study of the liver metastasis of pancreatic cancer cells suggested that endoplasmic reticulum aminopeptidase 1 (ERAP1)-secreting exosomes enhance the phagocytic capacity and NO synthesis activity of macrophages (89). However, some circulating exosomes can protect against phagocytosis by macrophage CD47 enrichment.

The effect of exosomes from non-metastatic K7 and Dunn osteosarcoma cells and the metastatic sublines K7M3 and DLM8 on macrophage phagocytosis was evaluated in a study of osteosarcoma lung metastasis (85). Exosomes secreted by the highly metastatic K7M3 and DLM8 cell lines were incubated with MHS mouse alveolar macrophages, which induced the mRNA expression of *IL-10*, *TGFB2*, and *CCL22* (markers of M2 macrophages). Reduced macrophage phagocytosis, exocytosis, and macrophage-mediated tumor cell killing were also observed. By contrast, exosomes from non-metastatic K7 or Dunn cells failed to inhibit macrophage phagocytosis, exocytosis, and cytotoxicity and did not induce increases in the mRNA expression of *IL10*, *TGFB2*, or *CCL22*.

The uptake of exosomes by macrophages is inseparable from clathrin-dependent endocytosis in which caveolin-1 is essential for the formation of pits (membrane depressions) and accumulates in membrane depressions (90). Clathrin protein heavy chain 1 (Cltc) is encoded by the *cltc* gene and is highly expressed in macrophages (91). When Cltc1 is knocked out, phagocytosis by monocyte-macrophages is inhibited.

### HCC Tumor-Derived exosomes are involved in the regulation of the polarization and function of macrophages

Liver macrophages can be activated to M1 and IL-13 *via* the classical activation pathway (bacterial lipopolysaccharide and interferon-gamma secreted by Th1 cells) and alternative activation pathways (cytokines IL-4, IL-10, and IL-13 secreted by Th2 cells). There are two subtypes of M2 macrophages, and these can be further subdivided into M2a, M2b, M2c, and M2d. M1-type macrophages mainly secrete pro-inflammatory factors, such as IL-12, IL-6, IL-18, IL-23, and tumor necrosis factor, and increase the expression of nitric oxide synthase, which is responsible for the defense against pathogen infection. The M2 type expresses high levels of IL-10, IL-1a/b inhibitor, mannose receptor (MRC1), arginase 1 (Arg1), and other anti-inflammatory factors. These two polarization modes are classic models for studies of macrophages (92, 93).

Immunity and metabolism are highly integrated and coordinated. In the initial stage of tissue hypoxia, the anaerobic glycolysis and pentose phosphate pathways of M1 macrophages are activated, whereas M2 macrophages mainly use oxidative phosphorylation and aerobic glycolysis to meet the energy requirements for tissue repair and remodeling. M1 macrophages are considered the most likely precursors of tumor-infiltrating macrophages, and TAMs are frequently M2 macrophages (94).

**Figure 2:**
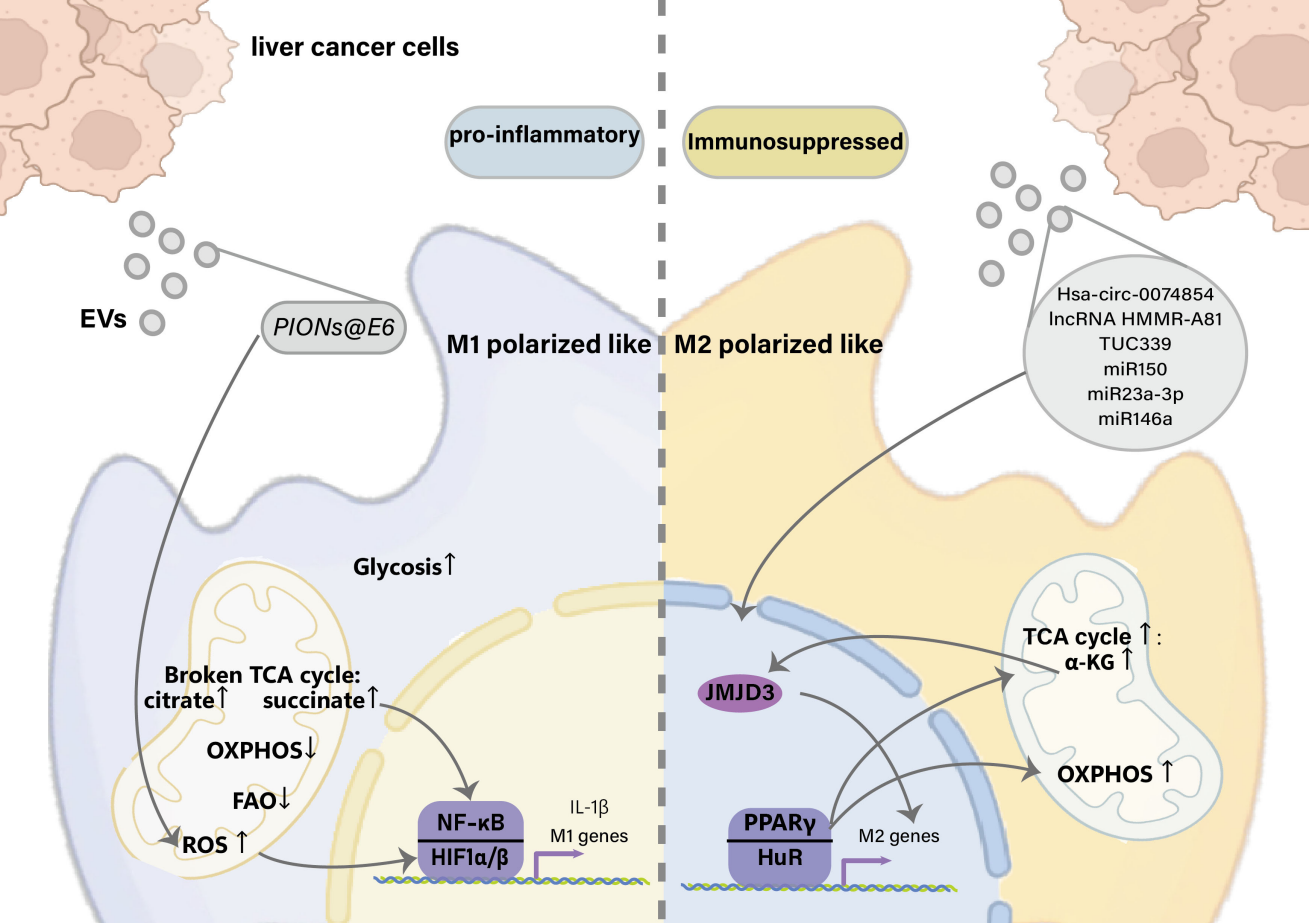
Effects of liver cancer-derived exosomes on macrophage polarization. EVs, Extracellular vesicles; TCA, tricarboxylic acid cycle; OXPHOS, Oxidative phosphorylation; FAO, Fatty Acid Oxidation; HIF1, hypoxia-inducible factor 1; NF-κB, Nuclear factor kappa-light-chain-enhancer of activated B cells; IL-1β, Interleukin 1 beta; α-KG, α-Ketoglutaric acid; JMJD3, Jumonji domain-containing protein-3; PPARγ, Peroxisome proliferator-activated receptor γ; HuR, Hu antigen R.

The long non-coding RNA (lncRNA) TUC339 is highly expressed in HCC-derived exosomes, which can be transferred across HCC cells to promote tumor growth and metastasis (55, 95). Furthermore, the exosomal long non-coding RNA (lncRNA) TUC339 can be transferred to neighboring macrophages to modulate M1/M2 polarization and suppress antitumor immune responses *in vitro*. Microarray studies have demonstrated that exosomal TUC339 downregulated TLR signaling and Fcγ receptor (FcγR)-mediated phagocytosis pathways in macrophages, and TUC339 knockdown increased the phagocytic activity of macrophages. TUC339 is also involved in cytokine and chemokine receptor signaling, although the exact mechanism is unclear. Tumor cell-derived exosomes also carry miRNAs that regulate the expression of immune response-related genes. miR150 is highly expressed in the plasma of patients with HCC and in HCC-derived exosomes and promotes the growth of vascular endothelial cells by secreting the TAM-derived cytokine factor VEGF (40). VEGF levels are reduced in the plasma and tumor tissues of tumor-bearing mice treated with miR150 inhibitors. HCC exosomal miR-23a-3p upregulates the programmed cell death ligand 1 (PD-L1) expression in macrophages *via* Signal transducer and activator of transcription 3 (STAT3) signaling, which significantly attenuates melatonin-treated HCC cell-derived exosomes (41). PD-L1 expression in phagocytes has been demonstrated *in vivo*. HCC-derived exosomes significantly increased CD11b+F4/80+CD206+ macrophages, accompanied by upregulation of M2-specific markers, including C-C chemokine ligand 17 (ccl17), C-C chemokine ligand 22 (ccl22), and arg-1. M2 polarization *in vitro* and in HCC-bearing mouse models is driven by miR146a, which is directly regulated by the zinc finger transcription factor Sal-like protein-4 (SALL4) in HCC cells (96, 97). The exosomal lncRNA HMMR-AS1 mediates macrophage polarization *via* the miR-147a/ARID3A axis under hypoxia and affects the progression of HCC (98) (**Figure 2**).

## Therapeutic prospects related to exosomes in liver cancer cells

### Role of H-TEXs in liver cancer drug resistance

In the TME, exosomes act as key regulators of the effects of chemotherapeutics by modulating drug efflux, epithelial–mesenchymal transition (EMT), autophagy phenotype, and immunosuppression (**Table 2**).

**Table 2:** The role of H-TEXs in liver cancer drug resistance.

Donor cells	Contents	Recipient cells	Functions	Mechanism	References
MHCC-97H	HGF	SMCC-7721	Induce sorafenib resistance *in vitro* and *in vivo*	HGF/cMET/Akt signaling	(99)
HepG2	linc-ROR	HepG2	Induce resistance to doxorubicin and camptothecin	Modulate TGF-β/Caspase 3/CD133 signaling	(100)
HepG2	linc-VLDLR	HepG2 and KMBC	Induce resistance to sorafenib and doxorubicin	Enhance ABCG2 expression	(101)
hepa1-6	Tumor-associated antigen	DCs	Increase sorafenib efficacy with PD-1 antibody	Regulate Treg accumulation *via* PD-1/PD-L1 pathway	(102)
HBV-infecte d HepG2	HBX	HepG2	Facilitate OXA resistance	Activate CMA pathway	(103)
AMSCs	miR-199a	HCC cells	Improve HCC chemosensitivity	mTOR pathway	(104)
HCC cells	circUHRF1	HCC cells	Anti-PD1 therapy resistance	NK cell dysfunction by upregulating TIM-3	(62)
HepG2	circ-SORE	HepG2	Induce resistance to sorafenib	Stabilize YBX1	(105)

### Exosome-related drug delivery system based on liver cancer therapy

Additionally, exosomes can assist in the early diagnosis of tumors, monitoring, and prognostic analyses. Because exosomes are endogenous vesicles, they benefit from low immunogenicity, good biodegradability, low toxicity, and the ability to cross the blood–brain barrier, making them good carriers for drug delivery (106). For example, using doxorubicin, Yong et al. (107) took advantage of the ability of tumor cells to efflux chemotherapeutic drugs through EVs to achieve chemoresistance and encapsulated porous silicon nanoparticles loaded with Adriamycin into tumor cell-derived exosomes. Liang et al. (108) demonstrated that tumor-repopulating cell (TRC)-derived three-dimensional (3D) extracellular microparticles (MPs), which benefit from their softness, accumulate substantially and readily penetrate the liver tumor parenchyma for efficient delivery of chemotherapeutic drugs into TRCs. This results in effective suicide-like TRC killing and favorable therapeutic outcomes. Cytospin-A-related softness of 3D-MPs plays an important role in regulating the *in vivo* transport process. These findings reveal a new aspect of MP biology and provide potentially effective strategies for drug delivery in cancer therapy. This Trojan horse-like nanodrug delivery system using tumor exosomes as a carrier has been shown to have a higher inhibitory effect on tumor cells *in vivo*, and the tail vein injection of tumor-bearing mice is comparable to that of doxorubicin alone. Greater enrichment was observed in the exosome-encapsulated doxorubicin-treated group than in the doxorubicin group. Similarly, the exosome chemotherapeutic drug loading method has been evaluated in research on glioma. However, the liver, the largest solid organ in the human body, contains the most tissue-resident macrophages, and KCs, which account for 80%–90% of all tissue macrophages in the body, are responsible for capturing and removing foreign bodies. Therefore, avoiding the capture of the mononuclear phagocytic cell system and ensuring the delivery of sufficient doses of drugs to the liver tumor area after entering the blood circulation are major issues that need to be resolved in cancer-targeted drug delivery research (109). Belhadj et al. (110) described an “eat/don’t-eat” decision switch for macrophages to evade phagocytosis by modifying CD47 outside of EVs. The effectiveness of this switch was verified by Du et al. (111). They engineered an exosome armed with three moieties, surface functionalization with CD47, membrane loading with ferroptosis inducer erastin, and core with photosensitizer RB. The exosomes displayed high delivery efficiency to tumors. Upon irradiation with a 532-nm laser in the tumor region, Erastin (Er) and Rose Bengal (RB) synergistically induced cell death.

### Exosome-based immunotherapy in HCC

Cancer immunotherapy reverses the immunosuppressive TME (112). Exosome-targeted immunotherapy of HCC is often associated with dendritic cell (DC)-derived exosomes (DEXs), which have great potential for immunotherapy applications (113, 114).

Lu et al. (115) infected a DC cell line (DC2.4), which was established by transfecting Granulocyte-macrophage colony-stimulating factor (GM-CSF) (Csf2), Myc, and Raf genes into C57BL/6 mice, with a lentivirus-expressing murine α-fetoprotein (AFP). They found that DC-AFP-derived exosomes (DEX-AFP) elicited strong antigen-specific immune responses, resulting in significantly delayed tumor growth and prolonged survival in various HCC mouse models (115). Zuo et al. (116) also used DEX as a carrier for a liver cancer vaccine to initiate a specific immune response against HCC. They decorated DEX with an HCC-targeting peptide (P47-P), an AFP epitope (AFP212-A2), and a functional domain of high-mobility group nucleosome-binding protein 1 (N1ND-N) and demonstrated its potential for the individualized treatment of HCC *via* universal DEX vaccines (116). Zhong et al. (117) enhanced the antitumor efficacy of a DEX vaccine for HCC using microwave ablation. Zuo et al. (118) demonstrated that alarmin-coated exosomes elicited durable large-scale antitumor immunity in mouse liver tumors. Shi et al. (102) showed that the combination of DC-TEX and a programmed cell death protein 1 (PD-1) antibody (Ab) enhances the efficacy of sorafenib.

However, immunotherapy clinical trials have shown that substantial work is still needed before these findings can be applied to the treatment of cancer in clinical settings. Despite the challenges, DEX remains a promising immunotherapeutic strategy. DEX acts as a stable vesicle with a long shelf life, and its immunostimulatory properties are easily manipulated (through donor DCs). Research advancements have expanded the use of DEX-based cancer treatments in clinical settings (119).

## Discussion

In this review, we evaluated the important role of exosomes in HCC progression and immunotherapy. Given that TAMs are a key component of the microenvironment, we summarize the regulatory mechanism by which liver cancer-derived exosomes regulate macrophage polarization, demonstrating that exosomes are a promising tool to target macrophages for HCC immunotherapy.

It should be emphasized that the interaction between the tumor and immunity is dynamic, heterogeneous, and bidirectional, including the immune response to drugs or external stimuli. Furthermore, the tumor cell state and even the genome are altered (120). Even so, the regulatory function of exosomes as messengers cannot be ignored, especially in the treatment of HCC. Chemotherapy resistance has become a major obstacle in improving the prognosis of patients. Targeting exosomes could be a promising strategy for reversing drug tolerance. In addition, the improvement of the efficacy of chemotherapy in patients with HCC by exosome anticancer drug delivery provides a new perspective for clinical treatment.

## Author contributions

Conceptualization, SY and LZ. Writing original manuscript, SY. Visualization, SY and JF. Writing review and editing, SY, JF,LX, YZ, JW, BL, JS and XY. All authors contributed to the article and approved the submitted version.

## Funding

National Natural Science Foundation of China (81672948); Jilin Provincial Research Foundation for Health Technology Innovation (2021JC034).

## Conflict of interest

The authors declare that the research was conducted in the absence of any commercial or financial relationships that could be construed as a potential conflict of interest.

## Publisher’s note

All claims expressed in this article are solely those of the authors and do not necessarily represent those of their affiliated organizations, or those of the publisher, the editors and the reviewers. Any product that may be evaluated in this article, or claim that may be made by its manufacturer, is not guaranteed or endorsed by the publisher.

## References

[1] Siegel RL Miller KD Fuchs HE Jemal A . Cancer statistics, 2022. CA Cancer J Clin (2022) 72(1):7–33. doi: 10.3322/caac.21708 35020204

[2] Chen Y Tian Z . HBV-induced immune imbalance in the development of HCC. Front Immunol (2019) 10:2048. doi: 10.3389/fimmu.2019.02048 31507621 PMC6718466

[3] Pandyarajan V Govalan R Yang JD . Risk factors and biomarkers for chronic hepatitis b associated hepatocellular carcinoma. Int J Mol Sci (2021) 22(2):479. doi: 10.3390/ijms22020479 PMC7825109 33418899

[4] Tian Z Hou X Liu W Han Z Wei L . Macrophages and hepatocellular carcinoma. Cell Biosci (2019) 9:79. doi: 10.1186/s13578-019-0342-7 31572568 PMC6761725

[5] Wen Y Lambrecht J Ju C Tacke F . Hepatic macrophages in liver homeostasis and diseases-diversity, plasticity and therapeutic opportunities. Cell Mol Immunol (2021) 18(1):45–56. doi: 10.1038/s41423-020-00558-8 33041338 PMC7852525

[6] Wu J Gao W Tang Q Yu Y You W Wu Z . M2 macrophage-derived exosomes facilitate HCC metastasis by transferring alphaM beta2 integrin to tumor cells. Hepatology (2021) 73(4):1365–80. doi: 10.1002/hep.31432 PMC8360085 32594528

[7] Guo Y Xiao Z Yang L Gao Y Zhu Q Hu L . Hypoxiainducible factors in hepatocellular carcinoma (Review). Oncol Rep (2020) 43(1):3–15. doi: 10.3892/or.2019.7397 31746396 PMC6908932

[8] Xu J Zhang J Zhang Z Gao Z Qi Y Qiu W . Hypoxic glioma-derived exosomes promote M2-like macrophage polarization by enhancing autophagy induction. Cell Death Dis (2021) 12(4):373. doi: 10.1038/s41419-021-03664-1 33828078 PMC8026615

[9] Bister N Pistono C Huremagic B Jolkkonen J Giugno R Malm T . Hypoxia and extracellular vesicles: A review on methods, vesicular cargo and functions. J Extracell Vesicles (2020) 10(1):e12002. doi: 10.1002/jev2.12002 33304471 PMC7710128

[10] Reinfeld BI Madden MZ Wolf MM Chytil A Bader JE Patterson AR . Cell programmed nutrient partitioning in the tumor microenvironment. Nature(2021) 593 (7858):282–88. doi: 10.1038/s41586-021-03442-1 33828302 PMC8122068

[11] Milane L Singh A Mattheolabakis G Suresh M Amiji MM . Exosome mediated communication within the tumor microenvironment. J Control Release (2015) 219:278–94. doi: 10.1016/j.jconrel.2015.06.029 26143224

[12] Sung S Kim J Jung Y . Liver-derived exosomes and their implications in liver pathobiology. Int J Mol Sci (2018) 19(12):3715. doi: 10.3390/ijms19123715 PMC6320937 30469540

[13] Jiao Y Xu P Shi H Chen D Shi H . Advances on liver cell-derived exosomes in liver diseases. J Cell Mol Med (2021) 25(1):15–26. doi: 10.1111/jcmm.16123 33247543 PMC7810930

[14] Nojima H Freeman CM Schuster RM Japtok L Kleuser B Edwards MJ . Hepatocyte exosomes mediate liver repair and regeneration *via* sphingosine-1-phosphate. J Hepatol (2016) 64(1):60–8. doi: 10.1016/j.jhep.2015.07.030 PMC4843792 26254847

[15] Conde-Vancells J Rodriguez-Suarez E Embade N Gil D Matthiesen R Valle M . Characterization and comprehensive proteome profiling of exosomes secreted by hepatocytes. J Proteome Res (2008) 7(12):5157–66. doi: 10.1021/pr8004887 PMC2696236 19367702

[16] Bukong TN Momen-Heravi F Kodys K Bala S Szabo G . Exosomes from hepatitis c infected patients transmit HCV infection and contain replication competent viral RNA in complex with Ago2-miR122-HSP90. PLos Pathog (2014) 10(10):e1004424. doi: 10.1371/journal.ppat.1004424 25275643 PMC4183590

[17] Chahal J Gebert LFR Gan HH Camacho E Gunsalus KC MacRae IJ . miR-122 and ago interactions with the HCV genome alter the structure of the viral 5' terminus. Nucleic Acids Res (2019) 47(10):5307–24. doi: 10.1093/nar/gkz194 PMC6547439 30941417

[18] Masaki T Arend KC Li Y Yamane D McGivern DR Kato T . miR-122 stimulates hepatitis c virus RNA synthesis by altering the balance of viral RNAs engaged in replication versus translation. Cell Host Microbe (2015) 17(2):217–28. doi: 10.1016/j.chom.2014.12.014 PMC4326553 25662750

[19] Yang Y Han Q Hou Z Zhang C Tian Z Zhang J . Exosomes mediate hepatitis b virus (HBV) transmission and NK-cell dysfunction. Cell Mol Immunol (2017) 14(5):465–75. doi: 10.1038/cmi.2016.24 PMC5423088 27238466

[20] Grunvogel O Colasanti O Lee JY Kloss V Belouzard S Reustle A . Secretion of hepatitis c virus replication intermediates reduces activation of toll-like receptor 3 in hepatocytes. Gastroenterology (2018) 154(8):2237–51 e16. doi: 10.1053/j.gastro.2018.03.020 29535029

[21] Harwood NM Golden-Mason L Cheng L Rosen HR Mengshol JA . HCV-infected cells and differentiation increase monocyte immunoregulatory galectin-9 production. J Leukoc Biol (2016) 99(3):495–503. doi: 10.1189/jlb.5A1214-582R 26475932 PMC6608045

[22] Zhang CY Yuan WG He P Lei JH Wang CX . Liver fibrosis and hepatic stellate cells: Etiology, pathological hallmarks and therapeutic targets. World J Gastroenterol (2016) 22(48):10512–22. doi: 10.3748/wjg.v22.i48.10512 PMC5192262 28082803

[23] Benbow JH Marrero E McGee RM Brandon-Warner E Attal N Feilen NA . Hepatic stellate cell-derived exosomes modulate macrophage inflammatory response. Exp Cell Res (2021) 405(1):112663. doi: 10.1016/j.yexcr.2021.112663 34051242 PMC8206040

[24] Li X Chen R Kemper S Brigstock DR . Extracellular vesicles from hepatocytes are therapeutic for toxin-mediated fibrosis and gene expression in the liver. Front Cell Dev Biol (2019) 7:368. doi: 10.3389/fcell.2019.00368 31998720 PMC6966099

[25] Chen L Charrier A Zhou Y Chen R Yu B Agarwal K . Epigenetic regulation of connective tissue growth factor by MicroRNA-214 delivery in exosomes from mouse or human hepatic stellate cells. Hepatology (2014) 59(3):1118–29. doi: 10.1002/hep.26768 PMC3943742 24122827

[26] Chen L Chen R Kemper S Charrier A Brigstock DR . Suppression of fibrogenic signaling in hepatic stellate cells by Twist1-dependent microRNA-214 expression: Role of exosomes in horizontal transfer of Twist1. Am J Physiol Gastrointest Liver Physiol (2015) 309(6):G491–9. doi: 10.1152/ajpgi.00140.2015 PMC4572411 26229009

[27] Wang H Lu Z Zhao X . Tumorigenesis, diagnosis, and therapeutic potential of exosomes in liver cancer. J Hematol Oncol (2019) 12(1):133. doi: 10.1186/s13045-019-0806-6 31815633 PMC6902437

[28] Conigliaro A Costa V Lo Dico A Saieva L Buccheri S Dieli F . CD90+ liver cancer cells modulate endothelial cell phenotype through the release of exosomes containing H19 lncRNA. Mol Cancer (2015) 14:155. doi: 10.1186/s12943-015-0426-x 26272696 PMC4536801

[29] Zeng Z Li Y Pan Y Lan X Song F Sun J . Cancer-derived exosomal miR-25-3p promotes pre-metastatic niche formation by inducing vascular permeability and angiogenesis. Nat Commun (2018) 9(1):5395. doi: 10.1038/s41467-018-07810-w 30568162 PMC6300604

[30] Han Q Zhao H Jiang Y Yin C Zhang J . HCC-derived exosomes: Critical player and target for cancer immune escape. Cells (2019) 8(6):558. doi: 10.3390/cells8060558 PMC6627799 31181729

[31] Li R Wang Y Zhang X Feng M Ma J Li J . Exosome-mediated secretion of LOXL4 promotes hepatocellular carcinoma cell invasion and metastasis. Mol Cancer (2019) 18(1):18. doi: 10.1186/s12943-019-0948-8 30704479 PMC6354392

[32] Gai X Tang B Liu F Wu Y Wang F Jing Y . mTOR/miR-145-regulated exosomal GOLM1 promotes hepatocellular carcinoma through augmented GSK-3beta/MMPs. J Genet Genomics (2019) 46(5):235–45. doi: 10.1016/j.jgg.2019.03.013 31186161

[33] Sun H Wang C Hu B Gao X Zou T Luo Q . Exosomal S100A4 derived from highly metastatic hepatocellular carcinoma cells promotes metastasis by activating STAT3. Signal Transduct Target Ther (2021) 6(1):187. doi: 10.1038/s41392-021-00579-3 34035222 PMC8149717

[34] Ye L Zhang Q Cheng Y Chen X Wang G Shi M . Tumor-derived exosomal HMGB1 fosters hepatocellular carcinoma immune evasion by promoting TIM-1(+) regulatory b cell expansion. J Immunother Cancer (2018) 6(1):145. doi: 10.1186/s40425-018-0451-6 30526680 PMC6288912

[35] Fu Q Zhang Q Lou Y Yang J Nie G Chen Q . Primary tumor-derived exosomes facilitate metastasis by regulating adhesion of circulating tumor cells *via* SMAD3 in liver cancer. Oncogene (2018) 37(47):6105–18. doi: 10.1038/s41388-018-0391-0 PMC6250679 29991801

[36] Jiang K Dong C Yin Z Li R Mao J Wang C . Exosome-derived ENO1 regulates integrin alpha6beta4 expression and promotes hepatocellular carcinoma growth and metastasis. Cell Death Dis (2020) 11(11):972. doi: 10.1038/s41419-020-03179-1 33184263 PMC7661725

[37] Dai W Wang Y Yang T Wang J Wu W Gu J . Downregulation of exosomal CLEC3B in hepatocellular carcinoma promotes metastasis and angiogenesis *via* AMPK and VEGF signals. Cell Commun Signal (2019) 17(1):113. doi: 10.1186/s12964-019-0423-6 31477130 PMC6721425

[38] Qiu QC Wang L Jin SS Liu GF Liu J Ma L . CHI3L1 promotes tumor progression by activating TGF-beta signaling pathway in hepatocellular carcinoma. Sci Rep (2018) 8(1):15029. doi: 10.1038/s41598-018-33239-8 30301907 PMC6177412

[39] Lee H-Y Chen C-K Ho C-M Lee S-S Chang C-Y Chen K-J . EIF3C-enhanced exosome secretion promotes angiogenesis and tumorigenesis of human hepatocellular carcinoma. Oncotarget (2018) 9(17):13193. doi: 10.18632/oncotarget.24149 29568350 PMC5862571

[40] Liu Y Zhao L Li D Yin Y Zhang CY Li J . Microvesicle-delivery miR-150 promotes tumorigenesis by up-regulating VEGF, and the neutralization of miR-150 attenuate tumor development. Protein Cell (2013) 4(12):932–41. doi: 10.1007/s13238-013-3092-z PMC4875402 24203759

[41] Liu J Fan L Yu H Zhang J He Y Feng D . Endoplasmic reticulum stress causes liver cancer cells to release exosomal miR-23a-3p and up-regulate programmed death ligand 1 expression in macrophages. Hepatology (2019) 70(1):241–58. doi: 10.1002/hep.30607 PMC6597282 30854665

[42] Fu X Liu M Qu S Ma J Zhang Y Shi T . Exosomal microRNA-32-5p induces multidrug resistance in hepatocellular carcinoma *via* the PI3K/Akt pathway. J Exp Clin Cancer Res (2018) 37(1):52. doi: 10.1186/s13046-018-0677-7 29530052 PMC5846230

[43] Fang T Lv H Lv G Li T Wang C Han Q . Tumor-derived exosomal miR-1247-3p induces cancer-associated fibroblast activation to foster lung metastasis of liver cancer. Nat Commun (2018) 9(1):191. doi: 10.1038/s41467-017-02583-0 29335551 PMC5768693

[44] Yokota Y Noda T Okumura Y Kobayashi S Iwagami Y Yamada D . Serum exosomal miR-638 is a prognostic marker of HCC *via* downregulation of VE-cadherin and ZO-1 of endothelial cells. Cancer Sci (2021) 112(3):1275–88. doi: 10.1111/cas.14807 PMC7935782 33426736

[45] Li W Xin X Li X Geng J Sun Y . Exosomes secreted by M2 macrophages promote cancer stemness of hepatocellular carcinoma *via* the miR-27a-3p/TXNIP pathways. Int Immunopharmacol (2021) 101(Pt A):107585. doi: 10.1016/j.intimp.2021.107585 34601333

[46] Kim HS Kim JS Park NR Nam H Sung PS Bae SH . Exosomal miR-125b exerts anti-metastatic properties and predicts early metastasis of hepatocellular carcinoma. Front Oncol (2021) 11:637247. doi: 10.3389/fonc.2021.637247 34386414 PMC8354570

[47] Zhang HY Liang HX Wu SH Jiang HQ Wang Q Yu ZJ . Overexpressed tumor suppressor exosomal miR-15a-5p in cancer cells inhibits PD1 expression in CD8+T cells and suppresses the hepatocellular carcinoma progression. Front Oncol (2021) 11:622263. doi: 10.3389/fonc.2021.622263 33816255 PMC8018596

[48] Lin XJ Fang JH Yang XJ Zhang C Yuan Y Zheng L . Hepatocellular carcinoma cell-secreted exosomal MicroRNA-210 promotes angiogenesis *In vitro* and in vivo. Mol Ther Nucleic Acids (2018) 11:243–52. doi: 10.1016/j.omtn.2018.02.014 PMC5992447 29858059

[49] Xue X Wang X Zhao Y Hu R Qin L . Exosomal miR-93 promotes proliferation and invasion in hepatocellular carcinoma by directly inhibiting TIMP2/TP53INP1/CDKN1A. Biochem Biophys Res Commun (2018) 502(4):515–21. doi: 10.1016/j.bbrc.2018.05.208 29859935

[50] Lin Q Zhou C-R Bai M-J Zhu D Chen J-W Wang H-F . Exosome-mediated miRNA delivery promotes liver cancer EMT and metastasis. Am J Trans Res (2020) 12(3):1080. PMC7137059 32269736

[51] Yang B Feng X Liu H Tong R Wu J Li C . High-metastatic cancer cells derived exosomal miR92a-3p promotes epithelial-mesenchymal transition and metastasis of low-metastatic cancer cells by regulating PTEN/Akt pathway in hepatocellular carcinoma. Oncogene (2020) 39(42):6529–43. doi: 10.1038/s41388-020-01450-5 PMC7561497 32917956

[52] Zhang Z Li X Sun W Yue S Yang J Li J . Loss of exosomal miR-320a from cancer-associated fibroblasts contributes to HCC proliferation and metastasis. Cancer Lett (2017) 397:33–42. doi: 10.1016/j.canlet.2017.03.004 28288874

[53] Zhou Y Ren H Dai B Li J Shang L Huang J . Hepatocellular carcinoma-derived exosomal miRNA-21 contributes to tumor progression by converting hepatocyte stellate cells to cancer-associated fibroblasts. J Exp Clin Cancer Res (2018) 37(1):324. doi: 10.1186/s13046-018-0965-2 30591064 PMC6307162

[54] Zhao S Li J Zhang G Wang Q Wu C Zhang Q . Exosomal miR-451a functions as a tumor suppressor in hepatocellular carcinoma by targeting LPIN1. Cell Physiol Biochem (2019) 53(1):19–35. doi: 10.33594/000000118 31162914

[55] Li X Lei Y Wu M Li N . Regulation of macrophage activation and polarization by HCC-derived exosomal lncRNA TUC339. Int J Mol Sci (2018) 19(10):2958. doi: 10.3390/ijms19102958 PMC6213212 30274167

[56] Wang D Xing N Yang T Liu J Zhao H He J . Exosomal lncRNA H19 promotes the progression of hepatocellular carcinoma treated with propofol *via* miR-520a-3p/LIMK1 axis. Cancer Med (2020) 9(19):7218–30. doi: 10.1002/cam4.3313 PMC7541143 32767662

[57] Wang J Pu J Zhang Y Yao T Luo Z Li W . Exosome-transmitted long non-coding RNA SENP3-EIF4A1 suppresses the progression of hepatocellular carcinoma. Aging (Albany NY) (2020) 12(12):11550. doi: 10.18632/aging.103302 32602848 PMC7343467

[58] Li B Mao R Liu C Zhang W Tang Y Guo Z . LncRNA FAL1 promotes cell proliferation and migration by acting as a CeRNA of miR-1236 in hepatocellular carcinoma cells. Life Sci (2018) 197:122–9. doi: 10.1016/j.lfs.2018.02.006 29421439

[59] Ma D Gao X Liu Z Lu X Ju H Zhang N . Exosome-transferred long non-coding RNA ASMTL-AS1 contributes to malignant phenotypes in residual hepatocellular carcinoma after insufficient radiofrequency ablation. Cell Prolif (2020) 53(9):e12795. doi: 10.1111/cpr.12795 32722884 PMC7507479

[60] Zhang H Deng T Ge S Liu Y Bai M Zhu K . Exosome circRNA secreted from adipocytes promotes the growth of hepatocellular carcinoma by targeting deubiquitination-related USP7. Oncogene (2019) 38(15):2844–59. doi: 10.1038/s41388-018-0619-z PMC6484761 30546088

[61] Wang G Liu W Zou Y Wang G Deng Y Luo J . Three isoforms of exosomal circPTGR1 promote hepatocellular carcinoma metastasis *via* the miR449a-MET pathway. EBioMedicine (2019) 40:432–45. doi: 10.1016/j.ebiom.2018.12.062 PMC6412851 30630697

[62] Zhang PF Gao C Huang XY Lu JC Guo XJ Shi GM . Cancer cell-derived exosomal circUHRF1 induces natural killer cell exhaustion and may cause resistance to anti-PD1 therapy in hepatocellular carcinoma. Mol Cancer (2020) 19(1):110. doi: 10.1186/s12943-020-01222-5 32593303 PMC7320583

[63] Chen W Quan Y Fan S Wang H Liang J Huang L . Exosome-transmitted circular RNA hsa_circ_0051443 suppresses hepatocellular carcinoma progression. Cancer Lett (2020) 475:119–28. doi: 10.1016/j.canlet.2020.01.022 32014458

[64] Hu K Li NF Li JR Chen ZG Wang JH Sheng LQ . Exosome circCMTM3 promotes angiogenesis and tumorigenesis of hepatocellular carcinoma through miR-3619-5p/SOX9. Hepatol Res (2021) 51(11):1139–52. doi: 10.1111/hepr.13692 34233088

[65] Zhang T Jing B Bai Y Zhang Y Yu H . Circular RNA circTMEM45A acts as the sponge of MicroRNA-665 to promote hepatocellular carcinoma progression. Mol Ther Nucleic Acids (2020) 22:285–97. doi: 10.1016/j.omtn.2020.08.011 PMC7516192 33230434

[66] Mo Z Liu D Rong D Zhang S . Hypoxic characteristic in the immunosuppressive microenvironment of hepatocellular carcinoma. Front Immunol (2021) 12:611058. doi: 10.3389/fimmu.2021.611058 33679749 PMC7928397

[67] Zhang B Tang B Gao J Li J Kong L Qin L . A hypoxia-related signature for clinically predicting diagnosis, prognosis and immune microenvironment of hepatocellular carcinoma patients. J Transl Med (2020) 18(1):342. doi: 10.1186/s12967-020-02492-9 32887635 PMC7487492

[68] Yaghoubi S Najminejad H Dabaghian M Karimi MH Abdollahpour-Alitappeh M Rad F . How hypoxia regulate exosomes in ischemic diseases and cancer microenvironment? IUBMB Life (2020) 72(7):1286–305. doi: 10.1002/iub.2275 32196941

[69] Yuen VW Wong CC . Hypoxia-inducible factors and innate immunity in liver cancer. J Clin Invest (2020) 130(10):5052–62. doi: 10.1172/JCI137553 PMC7524494 32750043

[70] Chen L Guo P He Y Chen Z Chen L Luo Y . HCC-derived exosomes elicit HCC progression and recurrence by epithelial-mesenchymal transition through MAPK/ERK signalling pathway. Cell Death Dis (2018) 9(5):513. doi: 10.1038/s41419-018-0534-9 29725020 PMC5938707

[71] Wang D Zhao C Xu F Zhang A Jin M Zhang K . Cisplatin-resistant NSCLC cells induced by hypoxia transmit resistance to sensitive cells through exosomal PKM2. Theranostics (2021) 11(6):2860–75. doi: 10.7150/thno.51797 PMC7806469 33456577

[72] Ju C Colgan SP Eltzschig HK . Hypoxia-inducible factors as molecular targets for liver diseases. J Mol Med (Berl) (2016) 94(6):613–27. doi: 10.1007/s00109-016-1408-1 PMC4879168 27094811

[73] Jafari R Rahbarghazi R Ahmadi M Hassanpour M Rezaie J . Hypoxic exosomes orchestrate tumorigenesis: molecular mechanisms and therapeutic implications. J Transl Med (2020) 18(1):474. doi: 10.1186/s12967-020-02662-9 33302971 PMC7731629

[74] Zhong L Liao D Li J Liu W Wang J Zeng C . Rab22a-NeoF1 fusion protein promotes osteosarcoma lung metastasis through its secretion into exosomes. Signal Transduct Target Ther (2021) 6(1):59. doi: 10.1038/s41392-020-00414-1 33568623 PMC7876000

[75] Bao MH Wong CC . Hypoxia, metabolic reprogramming, and drug resistance in liver cancer. Cells (2021) 10(7):1715. doi: 10.3390/cells10071715 34359884 PMC8304710

[76] Ageta H Tsuchida K . Post-translational modification and protein sorting to small extracellular vesicles including exosomes by ubiquitin and UBLs. Cell Mol Life Sci (2019) 76(24):4829–48. doi: 10.1007/s00018-019-03246-7 PMC11105257 31363817

[77] Wong CC Kai AK Ng IO . The impact of hypoxia in hepatocellular carcinoma metastasis. Front Med (2014) 8(1):33–41. doi: 10.1007/s11684-013-0301-3 24234682

[78] Lee JW Ko J Ju C Eltzschig HK . Hypoxia signaling in human diseases and therapeutic targets. Exp Mol Med (2019) 51(6):1–13. doi: 10.1038/s12276-019-0235-1 PMC6586801 31221962

[79] Anthony BJ Ramm GA McManus DP . Role of resident liver cells in the pathogenesis of schistosomiasis. Trends Parasitol (2012) 28(12):572–9. doi: 10.1016/j.pt.2012.09.005 23099112

[80] Davies LC Taylor PR . Tissue-resident macrophages: then and now. Immunology (2015) 144(4):541–8. doi: 10.1111/imm.12451 PMC4368161 25684236

[81] Craig AJ von Felden J Garcia-Lezana T Sarcognato S Villanueva A . Tumour evolution in hepatocellular carcinoma. Nat Rev Gastroenterol Hepatol (2020) 17(3):139–52. doi: 10.1038/s41575-019-0229-4 31792430

[82] Tacke F Zimmermann HW . Macrophage heterogeneity in liver injury and fibrosis. J Hepatol (2014) 60(5):1090–6. doi: 10.1016/j.jhep.2013.12.025 24412603

[83] Wang C Ma C Gong L Guo Y Fu K Zhang Y . Macrophage polarization and its role in liver disease. Front Immunol (2021) 12:803037. doi: 10.3389/fimmu.2021.803037 34970275 PMC8712501

[84] Shao J Li S Liu Y Zheng M . Extracellular vesicles participate in macrophage-involved immune responses under liver diseases. Life Sci (2020) 240:117094. doi: 10.1016/j.lfs.2019.117094 31760101

[85] Wolf-Dennen K Gordon N Kleinerman ES . Exosomal communication by metastatic osteosarcoma cells modulates alveolar macrophages to an M2 tumor-promoting phenotype and inhibits tumoricidal functions. Oncoimmunology (2020) 9(1):1747677. doi: 10.1080/2162402X.2020.1747677 32313728 PMC7153823

[86] Parada N Romero-Trujillo A Georges N Alcayaga-Miranda F . Camouflage strategies for therapeutic exosomes evasion from phagocytosis. J Adv Res (2021) 31:61–74. doi: 10.1016/j.jare.2021.01.001 34194832 PMC8240105

[87] Kanno S Hirano S Sakamoto T Furuyama A Takase H Kato H . Scavenger receptor MARCO contributes to cellular internalization of exosomes by dynamin-dependent endocytosis and macropinocytosis. Sci Rep (2020) 10(1):21795. doi: 10.1038/s41598-020-78464-2 33311558 PMC7733512

[88] Costa-Silva B Aiello NM Ocean AJ Singh S Zhang H Thakur BK . Pancreatic cancer exosomes initiate pre-metastatic niche formation in the liver. Nat Cell Biol (2015) 17(6):816–26. doi: 10.1038/ncb3169 PMC5769922 25985394

[89] Goto Y Ogawa Y Tsumoto H Miura Y Nakamura TJ Ogawa K . Contribution of the exosome-associated form of secreted endoplasmic reticulum aminopeptidase 1 to exosome-mediated macrophage activation. Biochim Biophys Acta Mol Cell Res (2018) 1865(6):874–88. doi: 10.1016/j.bbamcr.2018.03.009 29567213

[90] Simon L Campos A Leyton L Quest AFG . Caveolin-1 function at the plasma membrane and in intracellular compartments in cancer. Cancer Metastasis Rev (2020) 39(2):435–53. doi: 10.1007/s10555-020-09890-x PMC7311495 32458269

[91] Zhou X Liu X Yang X Wang L Hong Y Lian K . Tumor progress intercept by intervening in caveolin-1 related intercellular communication *via* ROS-sensitive c-myc targeting therapy. Biomaterials (2021) 275:120958. doi: 10.1016/j.biomaterials.2021.120958 34130142

[92] Saha S Shalova IN Biswas SK . Metabolic regulation of macrophage phenotype and function. Immunol Rev (2017) 280(1):102–11. doi: 10.1111/imr.12603 29027220

[93] Shapouri-Moghaddam A Mohammadian S Vazini H Taghadosi M Esmaeili SA Mardani F . Macrophage plasticity, polarization, and function in health and disease. J Cell Physiol (2018) 233(9):6425–40. doi: 10.1002/jcp.26429 PMC13482560 29319160

[94] Viola A Munari F Sanchez-Rodriguez R Scolaro T Castegna A . The metabolic signature of macrophage responses. Front Immunol (2019) 10:1462. doi: 10.3389/fimmu.2019.01462 31333642 PMC6618143

[95] Alzahrani FA El-Magd MA Abdelfattah-Hassan A Saleh AA Saadeldin IM El-Shetry ES . Potential effect of exosomes derived from cancer stem cells and MSCs on progression of DEN-induced HCC in rats. Stem Cells Int (2018) 2018:8058979. doi: 10.1155/2018/8058979 30224923 PMC6129855

[96] Yin C Han Q Xu D Zheng B Zhao X Zhang J . SALL4-mediated upregulation of exosomal miR-146a-5p drives T-cell exhaustion by M2 tumor-associated macrophages in HCC. Oncoimmunology (2019) 8(7):1601479. doi: 10.1080/2162402X.2019.1601479 31143524 PMC6527304

[97] Frundt T Krause L Hussey E Steinbach B Kohler D von Felden J . Diagnostic and prognostic value of miR-16, miR-146a, miR-192 and miR-221 in exosomes of hepatocellular carcinoma and liver cirrhosis patients. Cancers (Basel) (2021) 13(10):2484. doi: 10.3390/cancers13102484 34069692 PMC8161187

[98] Wang X Zhou Y Dong K Zhang H Gong J Wang S . Exosomal lncRNA HMMR-AS1 mediates macrophage polarization through miR-147a/ARID3A axis under hypoxia and affects the progression of hepatocellular carcinoma. Environ Toxicol (2022) 37(6):1357–72. doi: 10.1002/tox.23489 35179300

[99] Qu Z Wu J Wu J Luo D Jiang C Ding Y . Exosomes derived from HCC cells induce sorafenib resistance in hepatocellular carcinoma both *in vivo* and *in vitro* . J Exp Clin Cancer Res (2016) 35(1):159. doi: 10.1186/s13046-016-0430-z 27716356 PMC5045585

[100] Takahashi K Yan IK Kogure T Haga H Patel T . Extracellular vesicle-mediated transfer of long non-coding RNA ROR modulates chemosensitivity in human hepatocellular cancer. FEBS Open Bio (2014) 4:458–67. doi: 10.1016/j.fob.2014.04.007 PMC4050189 24918061

[101] Takahashi K Yan IK Wood J Haga H Patel T . Involvement of extracellular vesicle long noncoding RNA (linc-VLDLR) in tumor cell responses to chemotherapy. Mol Cancer Res (2014) 12(10):1377–87. doi: 10.1158/1541-7786.MCR-13-0636 PMC4201956 24874432

[102] Shi S Rao Q Zhang C Zhang X Qin Y Niu Z . Dendritic cells pulsed with exosomes in combination with PD-1 antibody increase the efficacy of sorafenib in hepatocellular carcinoma model. Transl Oncol (2018) 11(2):250–8. doi: 10.1016/j.tranon.2018.01.001 PMC5789129 29413757

[103] Liu DX Li PP Guo JP Li LL Guo B Jiao HB . Exosomes derived from HBV-associated liver cancer promote chemoresistance by upregulating chaperone-mediated autophagy. Oncol Lett (2019) 17(1):323–31. doi: 10.3892/ol.2018.9584 PMC6313222 30655770

[104] Lou G Chen L Xia C Wang W Qi J Li A . MiR-199a-modified exosomes from adipose tissue-derived mesenchymal stem cells improve hepatocellular carcinoma chemosensitivity through mTOR pathway. J Exp Clin Cancer Res (2020) 39(1):4. doi: 10.1186/s13046-019-1512-5 31898515 PMC6941283

[105] Xu J Ji L Liang Y Wan Z Zheng W Song X . CircRNA-SORE mediates sorafenib resistance in hepatocellular carcinoma by stabilizing YBX1. Signal Transduct Target Ther (2020) 5(1):298. doi: 10.1038/s41392-020-00375-5 33361760 PMC7762756

[106] Slomka A Mocan T Wang B Nenu I Urban SK Gonzales-Carmona M . EVs as potential new therapeutic Tool/Target in gastrointestinal cancer and HCC. Cancers (Basel) (2020) 12(10):3019. doi: 10.3390/cancers12103019 PMC7603109 33080904

[107] Yong T Zhang X Bie N Zhang H Zhang X Li F . Tumor exosome-based nanoparticles are efficient drug carriers for chemotherapy. Nat Commun (2019) 10(1):3838. doi: 10.1038/s41467-019-11718-4 31444335 PMC6707218

[108] Liang Q Bie N Yong T Tang K Shi X Wei Z . The softness of tumour-cell-derived microparticles regulates their drug-delivery efficiency. Nat BioMed Eng (2019) 3(9):729–40. doi: 10.1038/s41551-019-0405-4 31110292

[109] Yong T Wei Z Gan L Yang X . Extracellular vesicle-based drug delivery systems for enhanced anti-tumor therapies through modulating cancer-immunity cycle. Adv Mater (2022), e2201054. doi: 10.1002/adma.202201054 35726204

[110] Belhadj Z He B Deng H Song S Zhang H Wang X . A combined "eat me/don't eat me" strategy based on extracellular vesicles for anticancer nanomedicine. J Extracell Vesicles (2020) 9(1):1806444. doi: 10.1080/20013078.2020.1806444 32944191 PMC7480498

[111] Du J Wan Z Wang C Lu F Wei M Wang D . Designer exosomes for targeted and efficient ferroptosis induction in cancer *via* chemo-photodynamic therapy. Theranostics (2021) 11(17):8185–96. doi: 10.7150/thno.59121 PMC8344009 34373736

[112] Riley RS June CH Langer R Mitchell MJ . Delivery technologies for cancer immunotherapy. Nat Rev Drug Discovery (2019) 18(3):175–96. doi: 10.1038/s41573-018-0006-z PMC6410566 30622344

[113] Xu Z Zeng S Gong Z Yan Y . Exosome-based immunotherapy: a promising approach for cancer treatment. Mol Cancer (2020) 19(1):160. doi: 10.1186/s12943-020-01278-3 33183286 PMC7661275

[114] Pitt JM Andre F Amigorena S Soria JC Eggermont A Kroemer G . Dendritic cell-derived exosomes for cancer therapy. J Clin Invest (2016) 126(4):1224–32. doi: 10.1172/JCI81137 PMC4811123 27035813

[115] Lu Z Zuo B Jing R Gao X Rao Q Liu Z . Dendritic cell-derived exosomes elicit tumor regression in autochthonous hepatocellular carcinoma mouse models. J Hepatol (2017) 67(4):739–48. doi: 10.1016/j.jhep.2017.05.019 28549917

[116] Zuo B Zhang Y Zhao K Wu L Qi H Yang R . Universal immunotherapeutic strategy for hepatocellular carcinoma with exosome vaccines that engage adaptive and innate immune responses. J Hematol Oncol (2022) 15(1):46. doi: 10.1186/s13045-022-01266-8 35488312 PMC9052531

[117] Zhong X Zhou Y Cao Y Ding J Wang P Luo Y . Enhanced antitumor efficacy through microwave ablation combined with a dendritic cell-derived exosome vaccine in hepatocellular carcinoma. Int J Hyperthermia (2020) 37(1):1210–8. doi: 10.1080/02656736.2020.1836406 33100037

[118] Zuo B Qi H Lu Z Chen L Sun B Yang R . Alarmin-painted exosomes elicit persistent antitumor immunity in large established tumors in mice. Nat Commun (2020) 11(1):1790. doi: 10.1038/s41467-020-15569-2 32286296 PMC7156382

[119] Viaud S Thery C Ploix S Tursz T Lapierre V Lantz O . Dendritic cell-derived exosomes for cancer immunotherapy: What's next? Cancer Res (2010) 70(4):1281–5. doi: 10.1158/0008-5472.CAN-09-3276 20145139

[120] Nguyen PH Ma S Phua CZ Kaya NA Lai HL Lim CJ . Intratumoural immune heterogeneity as a hallmark of tumour evolution and progression in hepatocellular carcinoma. Nat Commun (2021) 12(1):1–13. doi: 10.1038/s41467-020-20171-7 33431814 PMC7801667

